# Fellow-Workers and the Roll of Honour

**Published:** 1914-11-07

**Authors:** 


### ROUND THE WORLD.

[The Editor Invites Early Information and Contributions Marked "Fellow-Workers."]

### Fellow-Workers and the Roll of Honour.

Lieutenant-Colonel Sir Joseph Fayrer, who has been doing excellent work as superintendent of the Edinburgh Royal Infirmary, is now in command of the 2nd Scottish General Hospital, which was established on mobilisation just outside Edinburgh in the Craigleith Poorhouse institution. Sir Joseph Fayrer succeeded to the baronetcy awarded to his distinguished father, the late Sir Joseph Fayrer, who for many years served with our Indian Forces, and in later life was an acknowledged authority on tropical diseases. He studied medicine at St. George's Hospital and Edinburgh, and took the qualifications of M.D. St. Andrews, F.R.C.S. Edin., and the triple Edinburgh and Glasgow diploma. A Knight of Grace of the Order of St. John of Jerusalem and honorary surgeon to the Cadet Corps, he was at one time medical officer to the Royal Horse Guards and the Duke of York's Military School at Chelsea.

Amongst the senior Scottish practitioners who are serving under Sir Joseph Fayrer may be mentioned Mr. J. M. Cotterill and Dr. C. W. Cathcart, who rank as lieutenant-colonels; also Dr. Boyd, Dr. J. D. Comrie, Dr. D. S. B. Thomson, and Dr. G. E. Wallace, who are majors in the 2nd Scottish General Hospital.

Mr. Joseph Montagu Cotterill is a well-known Edinburgh surgeon, president of the Edinburgh Medico-Chirurgical Society, and examiner in surgery to the Royal College of Surgeons there. An M.B., F.R.C.S. Edin., Mr. Cotterill has had a distinguished career as a prominent member of the staff of the Royal Infirmary, to which he was appointed consulting surgeon on his retirement from active operative work there.

Mr. Charles Walker Cathcart is the senior surgeon to the Edinburgh Royal Infirmary, and has for many years been prominent as a teacher in Edinburgh. He is an M.B. Edin., F.R.C.S. Eng., and F.R.C.S. Edin., and the author of several important works on surgical subjects.

Major John Dixon Comrie is in private life assistant physician to the Royal Infirmary, Edinburgh, and lecturer on the history of medicine at Edinburgh University. An M.D., F.R.C.P. Edin., he studied medicine also in London, Berlin, and Vienna. Dr. Comrie, who was at one time resident physician to the Royal Infirmary, is the editor of "Black's Medical Dictionary" and the author of many interesting articles on the history of medicine.

Major David Wallace, C.M.G., was surgeon in charge of the Edinburgh Hospital in South Africa during the Boer War, and was at that time mentioned in dispatches and gained a medal and clasp. An M.B., C.M., F.R.C.S. Edin., Major Wallace was successful in becoming elected to the surgical staff of the Royal Infirmary, Edinburgh, with which he has now been associated for some years. He is also honorary surgeon to the Victoria Hospital for Diseases of the Chest in Edinburgh, and lecturer and examiner in clinical surgery to the University there.

Among other eminent Edinburgh consultants who form the staff of the 2nd General Scottish Hospital on mobilisation, and who are liable to be called up for active service, may be mentioned Sir Robert William Philip, M.D., F.R.C.P. Edin., F.R.S.E., the well-known advocate and originator in this country of the dispensary system for dealing with tuberculosis. His present appointments include the responsible posts of senior physician to the Royal Infirmary, Edinburgh, and to the Royal Victoria Consumption Hospital there, as well as vice-chairman of the National Society for the Prevention of Consumption and lecturer and examiner in the University of Edinburgh. Sir Robert Philip, who is one of our foremost authorities on tuberculosis problems, is the author of "Pulmonary Tuberculosis" and "Public Aspects of the Prevention of Tuberculosis."

Colonel Michael W. H. Russell, who has succeeded Surgeon-General W. G. Macpherson as Deputy Director-General of the Army Medical Service, is an old Middlesex Hospital man, who qualified L.S.A. in 1881 and subsequently became an M.R.C.S. Eng. In early years he was house physician to the Middlesex Hospital and resident medical officer to the Royal United Hospital at Bath. Entering the Army Medical Department Colonel Russell has had a distinguished career therein for many years.

Dr. D. W. Carmalt-Jones, M.D., F.R.C.P., physician to Westminster Hospital, is now mobilised as captain in the R.A.M.C. Territorial Force and attached to the 8th Howitzer Brigade (2nd London Division). After spending some years at Oxford University Dr. Carmalt-Jones qualified from St. Mary's Hospital, where he held several resident and other appointments. Subsequently he was elected a member of the staff at Westminster, where he has since organised a department for therapeutic inoculation. He is also dean and joint-lecturer in medicine at Westminster Hospital Medical School, and was formerly senior house physician to the National Hospital for Paralysis and Epilepsy in Queen Square.

The medical staff of Lord Tredegar's yacht *Liberty* is entirely made up of St. Mary's men. Of these the principal medical officer, Dr. H. Gwynne Lawrence, is a well-known general physician in the West End whose *clientèle* includes an exceptional number of distinguished persons. Dr. Lawrence, whose father for many years carried on a large practice at Chepstow, gained an entrance scholarship when entering St. Mary's, and was subsequently awarded first-class honours with the gold medal and University scholarship in medicine, and first-class honours in obstetric medicine at the M.B. London examination.

Mr. W. H. Clayton-Greene, M.B., F.R.C.S., the chief surgeon, is on the staff of St. Mary's Hospital, and a popular teacher in the medical school attached thereto. From his earliest associations with St. Mary's, to which he proceeded from Cambridge with a University entrance scholarship, Mr. Clayton-Greene's activities have been distinguished by their brilliance and energy. At one time he was dean of the medical school, as well as lecturer in anatomy there, appointments which other demands on his time made it impossible for him to retain.

